# L'intérêt de l'accoutumance aux antituberculeux majeurs

**DOI:** 10.11604/pamj.2014.19.207.5086

**Published:** 2014-10-27

**Authors:** Sarra Aniked, Ouiam Bakouh, Jamal Eddine Bourkadi

**Affiliations:** 1 Service de Pneumo Phtisiologie, Hôpital Moulay Youssef, CHU Rabat, Maroc

**Keywords:** Hypersensibilité, antituberculeux, accoutumance, hypersensitivity, TB drugs, addiction

## Abstract

Les réactions d’ hypersensibilité aux antituberculeux sont relativement rares et graves par leur caractère imprévisible, elles conduisent généralement à l'arrêt ou au changement thérapeutique. Nous rapportons un cas d'hypersensibilité à trois antibacillaires majeurs (Isoniazide, Pyrazinamide, Ethombutol). Une accoutumance orale à ces trois médicaments a été réalisée permettant à la patiente de bénéficier d'un traitement antibacillaire optimal.

## Introduction

Le traitement antituberculeux est à l'origine de réactions d'hypersensibilité qui apparaissent chez 4 à 5% des patients, ces réactions sont graves pouvant dans certains cas mettre en jeu le pronostic vital. Pour épargner ce traitement, on a recours à l'accoutumance qui permet une induction de la tolérance aux médicaments.

## Patient et observation

**Figure 1:**
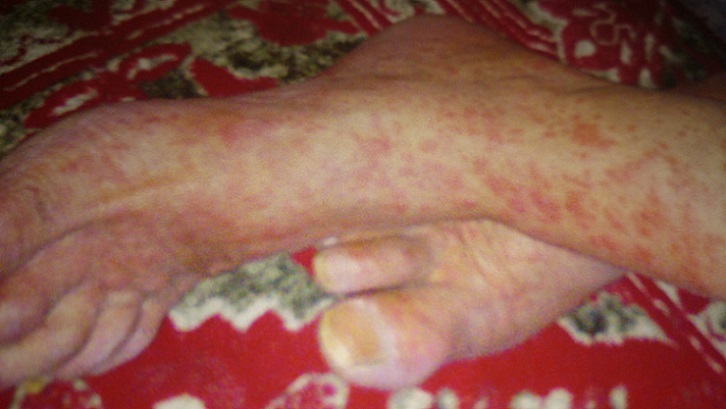
Lésions érythémateuses macculo papuleuse au niveau des deux pieds

Il s'agit d'une patiente âgée de 52 ans, sans antécédents pathologiques notables, mise sous traitement antituberculeux pour une tuberculose ganglionnaire confirmée histologiquement à base de 2 mois de Rifampicine 10 mg/kg/j, Isoniazide 5mg/kg/j, Pyrazinamide 25mg/kg/j, Ethombutol 20mg/kg/j, et 4 mois de rifampicine et isoniazide. Au 21^ème^ jour du traitement, la patiente a présenté une éruption cutanée macculo papuleuse généralisée sans atteinte des muqueuses, associée à un oedème du visage (Figure 1) et une fièvre chiffrée à 38.5°. L'examen clinique n'a pas trouvé une hépatosplénomégalie ou une adénopathie périphérique. Le bilan biologique a noté une hyper éosinophilie à 3570/mm^3^. Le traitement antituberculeux a été arrêté, et la patiente a été mise sous traitement antihistaminique et sous corticothérapie orale. La radio thoracique et l’échographie abdominale étaient sans anomalies, une biopsie cutanée revenue en faveur d'une toxidermie. Après 4 semaines, l’évolution a été marquée par la disparition complète des lésions cutanées et la normalisation du bilan biologique. Une réintroduction du traitement antituberculeux a été démarrée progressivement sous surveillance médicale (1^er^ jour: ¼ de la dose, 2^ème^ jour: ½ de la dose, 3^ème^ jour: dose complète). La réintroduction progressive de l'Ethombutol à la dose de 400 mg est suivie une heure après par l'apparition d'une éruption cutanée au niveau du visage et des deux membres supérieurs, cette symptomatologie est rapidement régressive sous antihistaminiques. La réintroduction progressive de la pyrazinamide à la dose de 400 mg est suivie une heure après également par la réapparition des lésions cutanées motivant l'arrêt de la pyrazinamide. Après la disparition des lésions cutanées, une réintroduction de l'isoniazide à la dose de 50 mg a été suivie une heure après par l'apparition de la même symptomatologie précédente.

Quelques jours après la disparition des lésions cutanées, une accoutumance séparée aux antibacillaires a été démarrée après l'accord de la patiente et sous surveillance médicale. Nous avons commencé par 1/1000000 (10^−6^) de la dose thérapeutique pour chaque médicament et nous avons doublé la concentration toutes les 30 mn (Tableau 1). L'accoutumance à l'isoniazide, à la rifampicine et au pyrazinamide s'est déroulée sans incidents. A 16 mg de l'ETB, la patiente a présenté un oedème du visage, des lésions érythémateuses généralisées, une hypotension et une fièvre, ce qui nous conduira à un arrêt définitif de l'ETB.

**Tableau 1:** Protocole d'accoutumance aux antituberculeux dans notre série

**Jour 1**		
	8:30	1×10^−6^ mg
	9:00	2×10^−6^ mg
	9:30	4×10^−6^ mg
	10:00	8×10^−6^ mg,
	10:30	16×10^−6^ mg
	11:00	32×10^−6^ mg,
	11:30	64×10^−6^ mg,
	12:00	128×10^−6^ mg,
	12:30	256×10^−6^ mg,
	13:00	512×10^−6^ mg
		La dose cumulée est 0.001mg
**Jour 2**		
	8:30	1×10^−3^mg,
	9:00	2×10^−3^mg,
	9:30	4×10^−3^mg,
	10:00	8×10^−3^mg,
	10:30	16×10^−3^mg,
	11:00	32×10^−3^ mg
	11:30	64×10^−3^mg,
	12:00	128×10^−3^mg,
	12:30	256×10^−3^mg,
	13:00	512×10^−3^ mg
		La dose cumulée est 1mg
**Jour 3**		
	8:30	1mg,
	9:00	2mg,
	9:30	4mg,
	10:00	8mg,
	10:30	16mg,
	11:00	32mg,
	11:30	64mg,
	12:00	128mg,
	12:30	256mg,
	13:00	512 mg
		La dose cumulée est 1023mg

## Discussion

La tuberculose est un problème majeur de santé publique dans le monde, son traitement repose essentiellement sur une quadrithérapie: Isoniazide (INH), Rifampicine (RIF), Pyrazinamide (PZA), et Ethombutol (ETB). Même si les effets secondaires de ce traitement sont multiples, les réactions d'hypersensibilité allergique sont assez rares (5% des cas), elle sont plus fréquentes chez les terrains immunodéprimés par le VIH (25% des cas), les insuffisants hépatiques et rénaux, et en cas d'un traitement administré d'une façon intermittente [1, 2]. Ormerod et al ont montré dans leur étude que ces réactions sont plus fréquentes chez les femmes que chez les hommes [2–4]. Tous les antituberculeux peuvent être incriminés, avec par ordre de fréquence le PZA, la streptomycine (STP), l'ETB, la RIF, et l'INH [5]. Les réactions d’;hypersensibilité aux antituberculeux surviennent souvent dans les deux premiers mois du traitement [2], il s'agit d'allergies cutanées dans 60% des cas [5].

Le diagnostic de certitude d'une réaction allergique aux antituberculeux peut être porté par les tests cutanés réalisés en milieu spécialisé sous forme de prick test ou d’;intradermoréaction [2]. La place des examens biologiques dans le diagnostic des allergies médicamenteuses est limitée, certaines explorations biologiques peuvent orienter vers un mécanisme immunopathogénique (hystaminémie, tryptasémie). Le dosage de l'IgE spécifique pourra dans l'avenir être une alternative diagnostique [6].

La réintroduction progressive des antituberculeux, sous surveillance médicale, en commençant par le médicament le moins incriminé semble être un outil diagnostique et thérapeutique [2]. Dans notre cas, les manifestations cliniques réapparaissent après l'introduction séparée de chaque médicament antituberculeux.

Devant l'hypersensibilité à deux ou plus des antituberculeux majeurs, une accoutumance aux antituberculeux s'impose. Cette méthode est le plus souvent efficace quelque soit le médicament en cause. Plusieurs protocoles d'accoutumance ont été décrits: C.L Holland et al et J. Matz et al ont décrit un protocole d'accoutumance rapide [7, 8], I Drira et al ont réalisé une accoutumance lente au rifater sur 21 jours [9]. Le protocole de Demoly a été publié pour d'autres médicaments, il a débuté la désensibilisation par 1/1000000 de la dose thérapeutique. L'accoutumance aux formes combinées pose un problème d'identification du produit en cause, elle entraine fréquemment des effets indésirables, J Drira et A Meybeck ont rapporté un cas d'accoutumance rapide par voie orale au Rifater menée avec succès [1–9].

Les résultats des différents protocoles de l'accoutumance sont généralement favorables, le taux de succès varie entre 75% pour l’;isoniazide et 82% pour la rifampicine [2].

## Conclusion

Les réactions allergiques aux antituberculeux sont graves. Selon notre expérience et dans la majorité des cas, l'accoutumance par voie orale des antibacillaires se déroule avec succès permettant la reprise du médicament en cause.
